# RNA sequence analysis of rat acute experimental pancreatitis with and without fatty liver: a gene expression profiling comparative study

**DOI:** 10.1038/s41598-017-00821-5

**Published:** 2017-04-07

**Authors:** Qian Wang, Hongkai Yan, Gang Wang, Zhaoyan Qiu, Bin Bai, Shiqi Wang, Pengfei Yu, Quanxin Feng, Qingchuan Zhao, Xianli He, Chaoxu Liu

**Affiliations:** 1 Department of General Surgery, Tangdu Hospital, Fourth Military Medical University, Xi’an, 710038 China; 2 Department of General Surgery, Huashan Hospital, Fudan University, Shanghai, 201907 China; 3 Department of General Surgery, The General Hospital of the People’s Liberation Army, Beijing, 100039 China; 4 Department of Surgery, Xijing Hospital of Digestive Diseases, Fourth Military Medical University, Xian, 710032 China

## Abstract

Fatty liver (FL) is one of the risk factors for acute pancreatitis and is also indicative of a worse prognosis as compared to acute pancreatitis without fatty liver (AP). The aim of the present study was to analyze, at the hepatic level, the differentially expressed genes (DEGs) between acute pancreatitis with fatty liver (APFL) rats and AP rats. GO (Gene Ontology) and KEGG (Kyoto Encyclopedia of Genes and Genomes) pathway analyses of these DEGs indicated that PPARα signalling pathway and fatty acid degradation pathway may be involved in the pathological process of APFL, which indicated that fatty liver may aggravate pancreatitis through these pathways. Moreover, the excessive activation of JAK/STAT signaling pathway and toll-like receptor signaling pathway was also found in APFL group as shown in heat map. In conclusion, the inhibition of PPARα signaling pathway and the fatty acid degradation pathway may lead to the further disorder of lipid metabolism, which can aggravate pancreatitis.

## Introduction

Acute pancreatitis (AP) is an acute inflammatory process of the pancreas, with wide clinical variation, ranging from mild discomfort to severe systemic complications^1^. Mild AP has a self-limiting course with a low mortality rate. It responds to conservative treatment and patients recover within a few days. In contrast, severe AP may lead to a mortality rate of 10–24%^2–4^.

Several risk factors for AP have been reported, including alcohol^5^, gallstones, smoking^6, 7^, obesity^8^, and non-alcoholic fatty liver disease (NAFLD)^9^. NAFLD is a clinical term that refers to excess fat deposition in the liver without excessive alcohol intake^10^. This is an increasing worldwide disease, which encompasses a wide spectrum of complications, ranging from simple steatosis to cirrhosis and hepatocellular carcinoma^11^. Our previous studies have shown that the prognosis of pancreatitis patients with a fatty liver disease was more severe than those with non-fatty liver disease^12^. Although much effort has been made to understand the underlying mechanism, the current knowledge remains limited. Therefore, the primary purpose of this study was to explore the differences between APFL and AP and make a primary exploration on the possible effects of fatty liver on pancreatitis.

Recently, next-generation sequencing technology has had a profound impact on a broad range of biological applications. RNA sequencing (RNA-seq) is a promising and widely used technology for analysis of the complete characterization of RNA transcripts, including gene fusion detection and transcription start site mapping^13^. In the present study, we used, for the first time, RNA-seq method to analyze the DEGs between APFL and AP rats. Next, the DEGs were mapped to terms in the GO database and were subjected to KEGG pathways enrichment analysis to determine the functions of these dysregulated genes. Hence, this study would enhance our understanding of the different mechanism between APFL and AP, which may provide some clues to the identification of potential therapeutic targets.

## Results

### Overall experimental procedure

**Figure 1:**
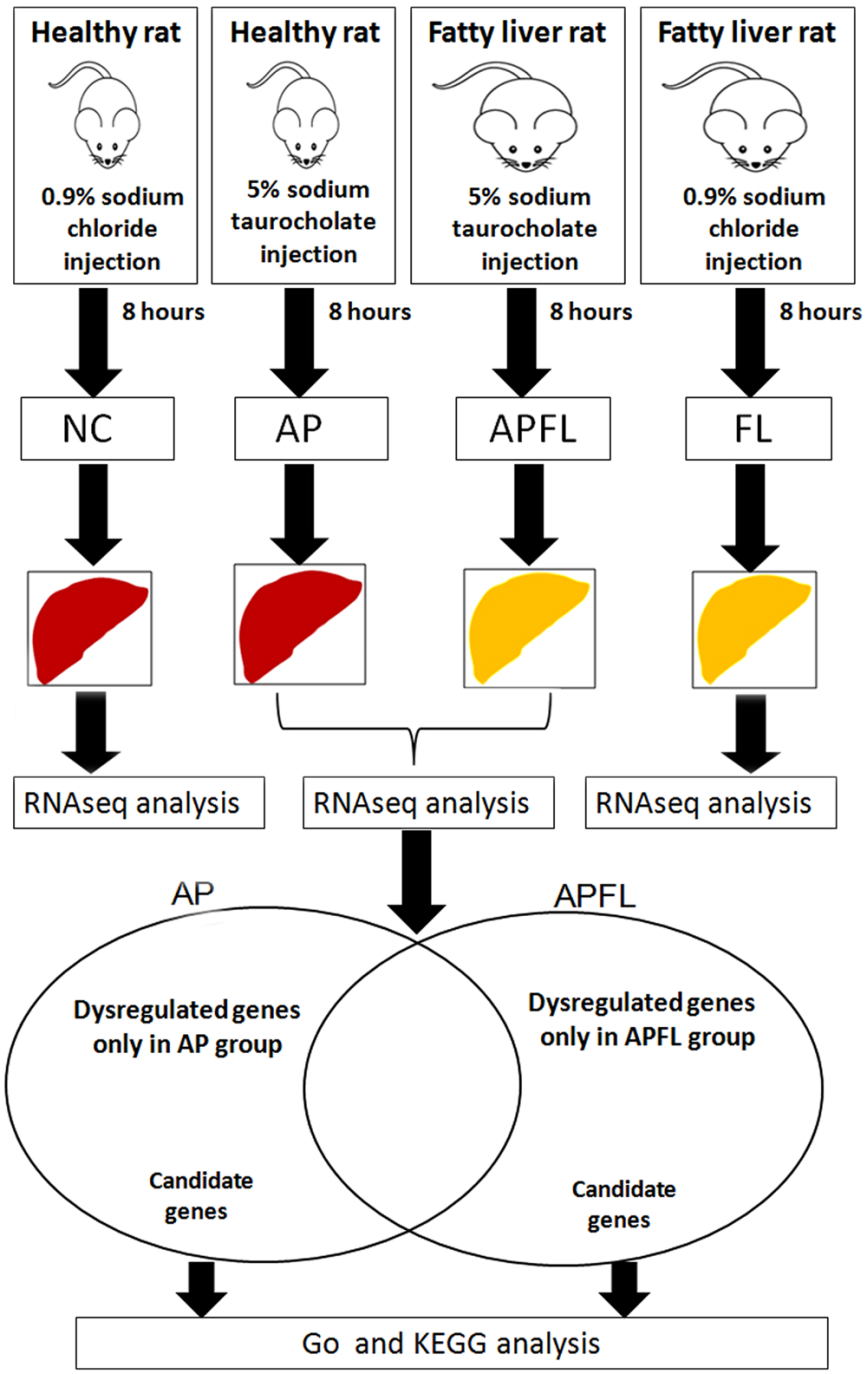
Experimental flow graph. Step 1: Model induction and liver collection; Step 2: RNA-seq analysis of DEGs between APFL and AP group; Step 3: GO and KEGG analysis of DEGs between APFL and AP.

Healthy SD rats were randomly divided into two groups. One group of 5% sodium taurocholate retrograde pancreatic duct injection was as acute pancreatitis group (AP), another group of intraperitoneal injection of 0.9% saline was as control group (NC); Fatty liver SD rats were randomly divided into two groups. One group of 5% sodium taurocholate retrograde pancreatic duct injection was as acute pancreatitis with fatty liver group (APFL), another group of intraperitoneal injection of 0.9% saline was as fatty liver group (FL). Eight hours later, the liver of each rat was collected for RNA-seq analysis. In order to explore the difference in pathogenesis between APFL and AP, we compared the differentially expressed genes between them. The specific experimental design is shown in Fig. 1.

### Diet-induced fatty liver and acute pancreatitis induction

**Figure 2:**
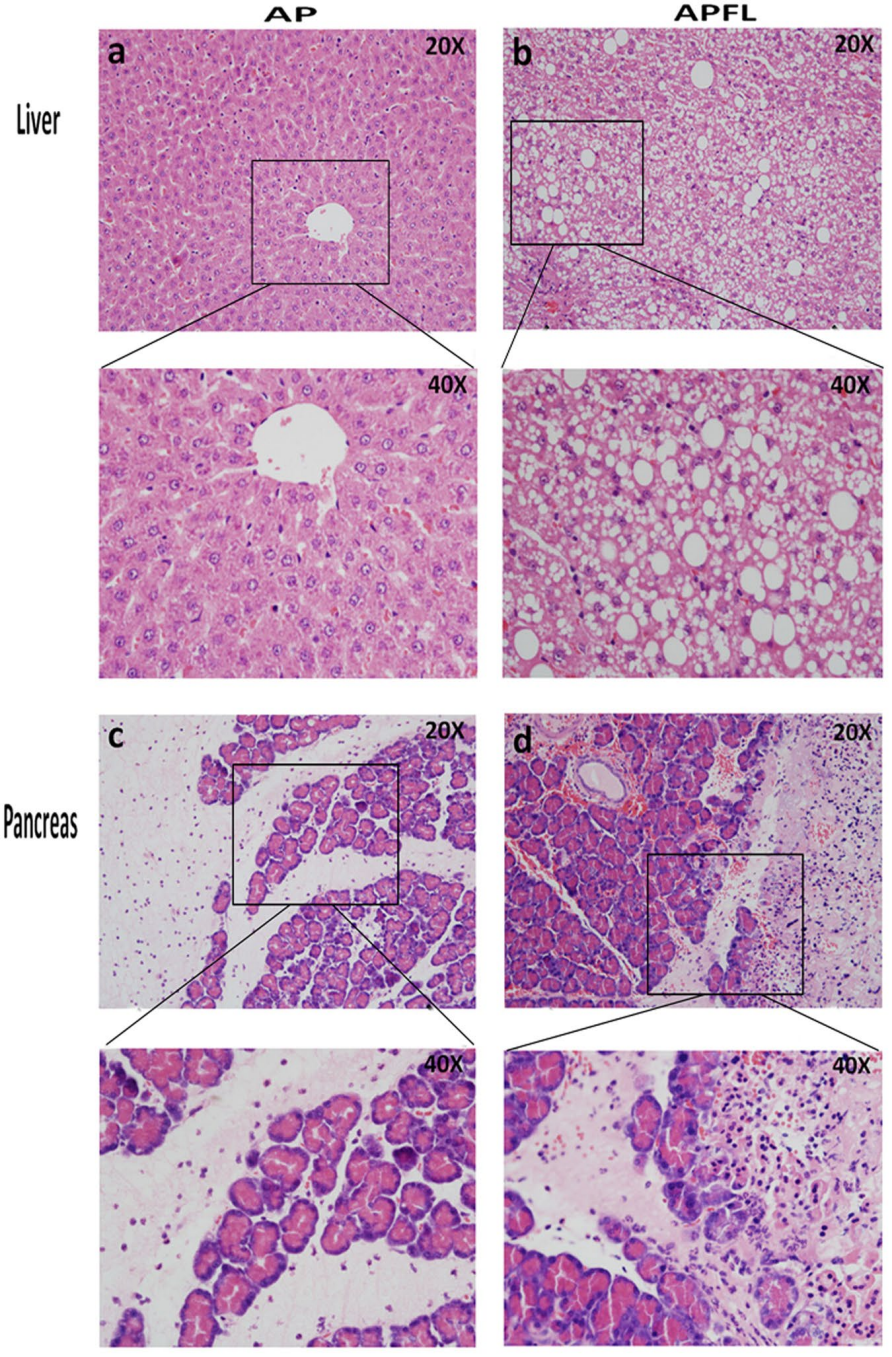
Tissue morphology in APFL and AP models. H&E staining: liver structure of rats in AP group (**a**) and APFL group (**b**); pancreas structure in AP (**c**) and APFL (**d**) group.

According to the percentage of fatty degeneration of hepatic parenchymal cells, simple fatty liver can be divided into the following 4 degrees: F0: <5% fatty degeneration of liver cells; F1: 5–30% fatty degeneration of liver cells; F2: 30–50% fatty degeneration of liver cells; F3: 50–75% fatty degeneration of liver cells; F4: above 75% fatty degeneration of liver cells. As shown on H&E staining slides, the liver cells of rats in high-fat diet group were obviously swollen, and vacuolar lipid droplets were observed in the cytoplasm of hepatocytes, indicating the establishment of fatty liver. The rate of fatty degeneration of liver cells was above 75 percent and there was no significant inflammatory cell infiltration and fibrosis in the liver lobule. Therefore, the stage of the fatty liver in this model was a simple steatosis (F4 degree) (Fig. 2b). The structure of normal liver was clear and complete (Fig. 2a). Compared with AP group, the pancreas of APFL exhibited more severe edema, inflammatory infiltration and acinar necrosis after establishment of acute pancreatitis (Fig. 2c,d).

### Sequencing evaluation

The RNA quality reached the sequencing requirement. The percentages of reads containing N, adaptors, clean reads and low-quality reads were calculated, and more than 97% of the raw reads passed the filter in each sample (Supplementary Fig. S1a). To confirm whether the number of detected genes increases proportionally to sequencing amount (total clean reads number), saturation analysis was performed. The results showed that the number of detected genes tended to saturation (Supplementary Fig. S1b). During preparation of the cDNA sequencing libraries, the mRNA was first fragmented into short segments by chemical methods and then sequenced. We used the distribution of read location on the genes to evaluate the randomness of breaking. In this study, the evenly distributed reads in every position of the genes indicated that the randomness of breaking of these samples was good (Supplementary Fig. S1c). Gene coverage was calculated as the percentage of a gene covered by reads from each sample. Supplementary Fig. S1d showed the distribution gene coverage of all the samples. Approximately 50% of total genes had coverage between 90–100%. To evaluate the result reliability, the correlation between replicates was calculated. Supplementary Fig. S1e shows the correlation of two replicates; the pearson correlation, which was closed to 1, indicated the good repeatability of the experiments. These results provide a favorable reliability for the quantitative analysis of DEGs.

### Analysis of differential gene expression

**Figure 3:**
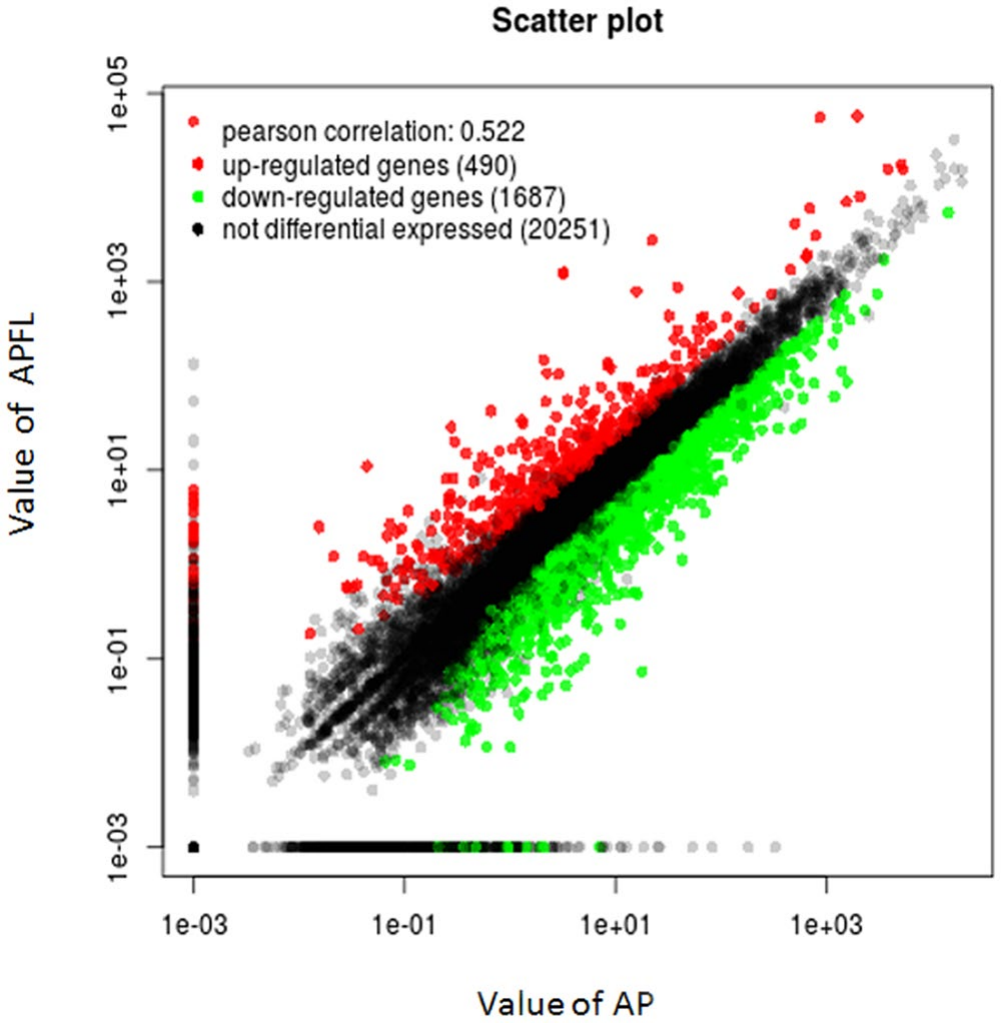
Differentially expressed genes between APFL and AP. Red spots represented up-regulated genes, and green spots down-regulated genes. Black spots indicate genes that were not differentially expressed between the two samples.

The scatter diagram results showed significantly differentially expressed genes (DEGs) between APFL and AP. A total of 2177 unigenes showed significant differential expression (false discovery rate [FDR] ≤ 0.001, |log2 ratio| ≥ 1). Among these unigenes, 490 genes were up-regulated and 1687 genes were down-regulated (Fig. 3). DEGs are partly shown in shown in Table 1.

**Table 1:** List of representative dysregulated genes between APFL and AP.

Gene name	Description	Log_2_ (Fold change)	Functions	Significance (P-value)
		Log_2_ (APFL/AP)		
RT1-A3	Uncharacterized protein	18.07504	Positive regulation of T cell mediated cytotoxicity	Yes (1.32E-05)
MSC	Musculin	12.34538	Negative regulation of transcription from RNA polymerase II promoter	Yes (9.45E-04)
FBXO27	F-box protein 27	11.24744	Glycoprotein binding	Yes (1.76E-07)
MSX1	Msh homeobox 1	10.72489	Negative regulation of cell growth	Yes (3.39E-04)
RGD1561212	Similar to RIKEN cDNA	10.69475	Exhibits retinoic acid receptor binding	Yes (1.46E-07)
SIGLEC8	Sialic acid binding Ig-like lectin 8	9.65489	Intracellular signal transduction	Yes (2.15E-05)
RAMP3	Receptor (G protein-coupled) activity modifying protein 3	9.49772	Regulation of G-protein coupled receptor protein signaling pathway	Yes (3.41E-18)
ZFYVE28	Zinc finger, FYVE domain containing 28	9.45763	Negative regulation of epidermal growth factor	Yes (7.88E-09)
TNFAIP6	Tumor necrosis factor alpha induced protein 6	9.33946	Negative regulation of inflammatory response	Yes (1.36E-06)
AOC1	Amine oxidase, copper containing 1	9.18586	Amine metabolic process	Yes (1.03E-04)
TNN	Tenascin N	8.82202	Cell-matrix adhesion	Yes (4.54E-07)
PTX3	Pentraxin 3	8.80702	Innate immune response	Yes (1.73E-06)
LMX1A	LIM homeobox transcription factor 1 alpha	8.36729	Regulation of transcription, DNA-templated	Yes (1.65E-04)
RASEF	RAS and EF hand domain containing	7.90165	Rab protein signal transduction	Yes (1.77E-04)
LOC497963	Similar to Nitric oxide synthase	7.78913	Nitric oxide biosynthetic process	Yes (2.15E-03)
DGKH	Diacylglycerol kinase	7.55722	Protein oligomerization	Yes (4.43E-06)
SLC7AL1	Solute carrier family 7, member 11	7.18928	Response to oxidative stress	Yes (7.32E-06)
DNM3	Dynamin 3	5.22016	Anatomical structure development	Yes (1.46E-07)
LRRC8E	Leucine rich repeat containing 8 family	5.07676	Ion transport	Yes (2.36E-06)
VWA2	Von Willebrand factor A domain containing 2	4.13783	Regulation of insulin receptor signaling pathway	Yes (2.53 E-03)
EGR2	Early growth response 2	4.11453	Cellular protein modification process	Yes (5.49 E-06)
FOSL2	Fos-like antigen 2	4.03226	Positive regulation of fibroblast proliferation	Yes (1.49 E-03)
TREM1	Triggering receptor expressed on myeloid cells 1	3.94884	Neutrophil chemotaxis	Yes (3.92 E-03)
FOSL1	Fos-like antigen 1	3.30191	Neurological system process	Yes (1.80 E-05)
GAS1	Growth arrest-specific 1	2.79755	Negative regulation of mitotic cell cycle	Yes (1.10E-20)
WDR4	WD repeat domain 4	1.30914	tRNA methylation	Yes (8.61E-03)
PTPRM	Protein tyrosine phosphatase, receptor type, M	−1.0036	Peptidyl-tyrosine dephosphorylation	Yes (1.55 E-04)
CDKN1C	CDKI protein long isoform	−1.71620	Cell cycle arrest	Yes (6.87 E-03)
UBD	Ubiquitin D	−2.32347	Protein ubiquitination	Yes (2.83E-06)
RGMA	Repulsive guidance molecule family member A	−2.87523	Negative regulation of collateral sprouting	Yes (8.81E-19)
BRDT	Bromodomain, testis-specific	−2.90388	Chromatin remodeling	Yes (4.13 E-03)
CD8A	CD8a molecule	−3.37913	Response to stress	Yes (6.91E-06)
FOXH1	Forkhead box H1	−3.95036	Anatomical structure development	Yes (9.49E-23)
PDE3A	Phosphodiesterase 3A	−4.67631	Small molecule metabolic process	Yes (4.37E-06)
TFF3	Tff3 molecule	−4.82677	Regulation of glucose metabolic process	Yes (5.44E-04)
OTOGL	Otogelin-like	−6.85145	Sensory perception of sound	Yes (6.46 E-04)
NPR3	Natriuretic peptide receptor 3	−8.79741	Negative regulation of adenylate cyclase activity	Yes (1.81E-03)
LOC100360055	Cytochrome P450 2B15-like	−9.82802	Xenobiotic metabolic process	Yes (8.93E-05)
MMD2	Monocyte to macrophage differentiation -associated 2	−10.26003	Protein phosphorylation	Yes (2.30E-08)
CML3	Camello-like 3	−10.90368	Gastrulation with mouth forming second	Yes (1.13E-13)
ALDH1A7	Aldehyde dehydrogenase family 1	−13.13943	Oxidation-reduction process	Yes (3.74E-28)

### Gene ontology (GO) classification of DEGs

**Figure 4:**
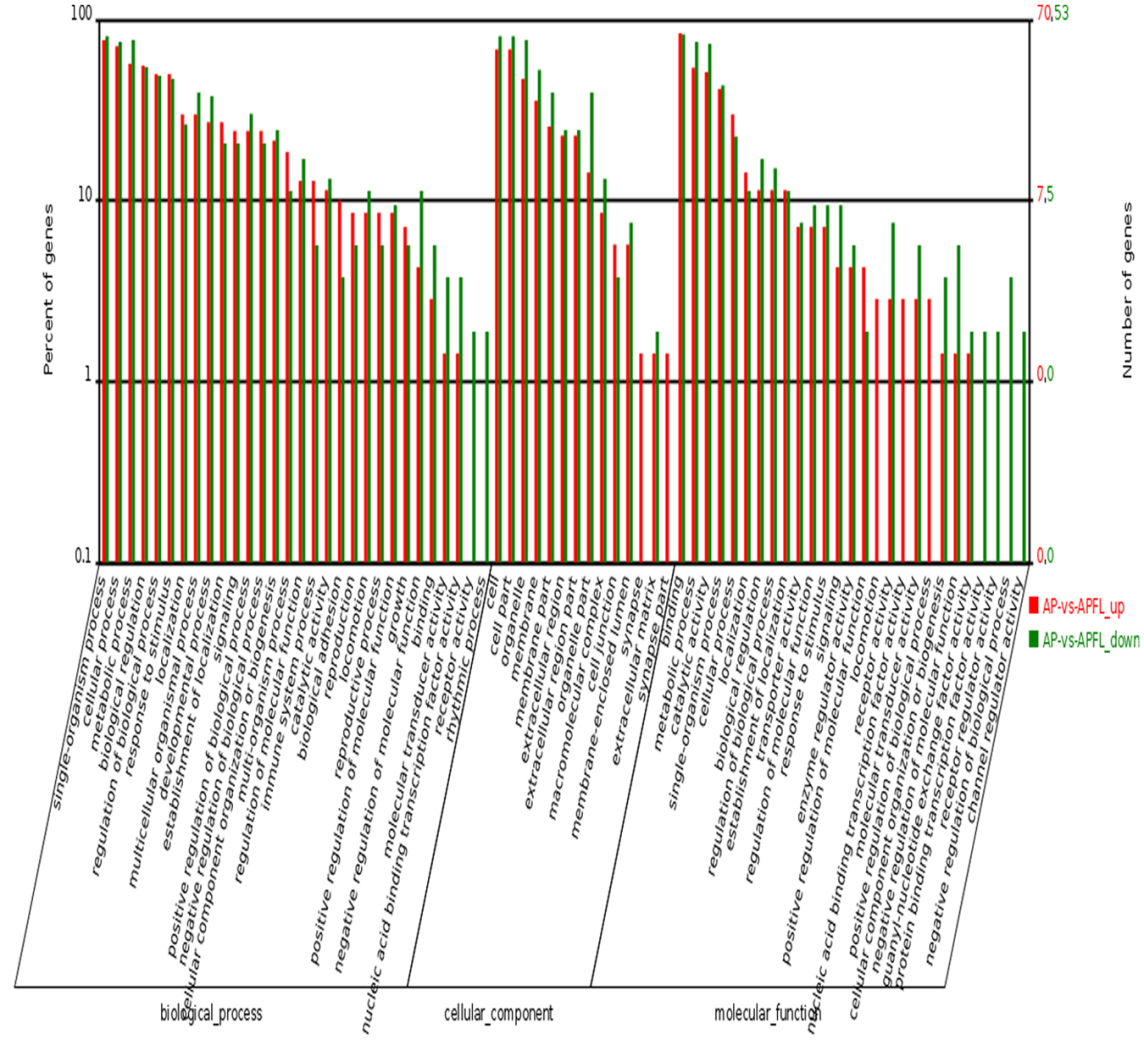
GO classification of DEGs between APFL and AP. The x-axis indicated the subcategories, the left y-axis represented the percentage of a specific category of DEGs and the right y-axis indicated the number of DEGs.

To determine the function of differentially expressed genes, all DEGs were mapped to terms in the GO database. A total of 2177 DEGs between APFL and AP samples were categorized into the three main categories of GO classification (e.g., biological process, cellular component and molecular function). For molecular function category, metabolic process, catalytic activity, and cofactor binding were the top abundant subcategories. Under the cellular component category, a large number of up-regulated, as well as down-regulated DEGs were categorized as cell part, cell and organelle. For biological processes, most of those were classified into cellular process and single-organism process (Fig. 4).

### KEGG pathway analysis of DEGs between APFL and AP

**Figure 5:**
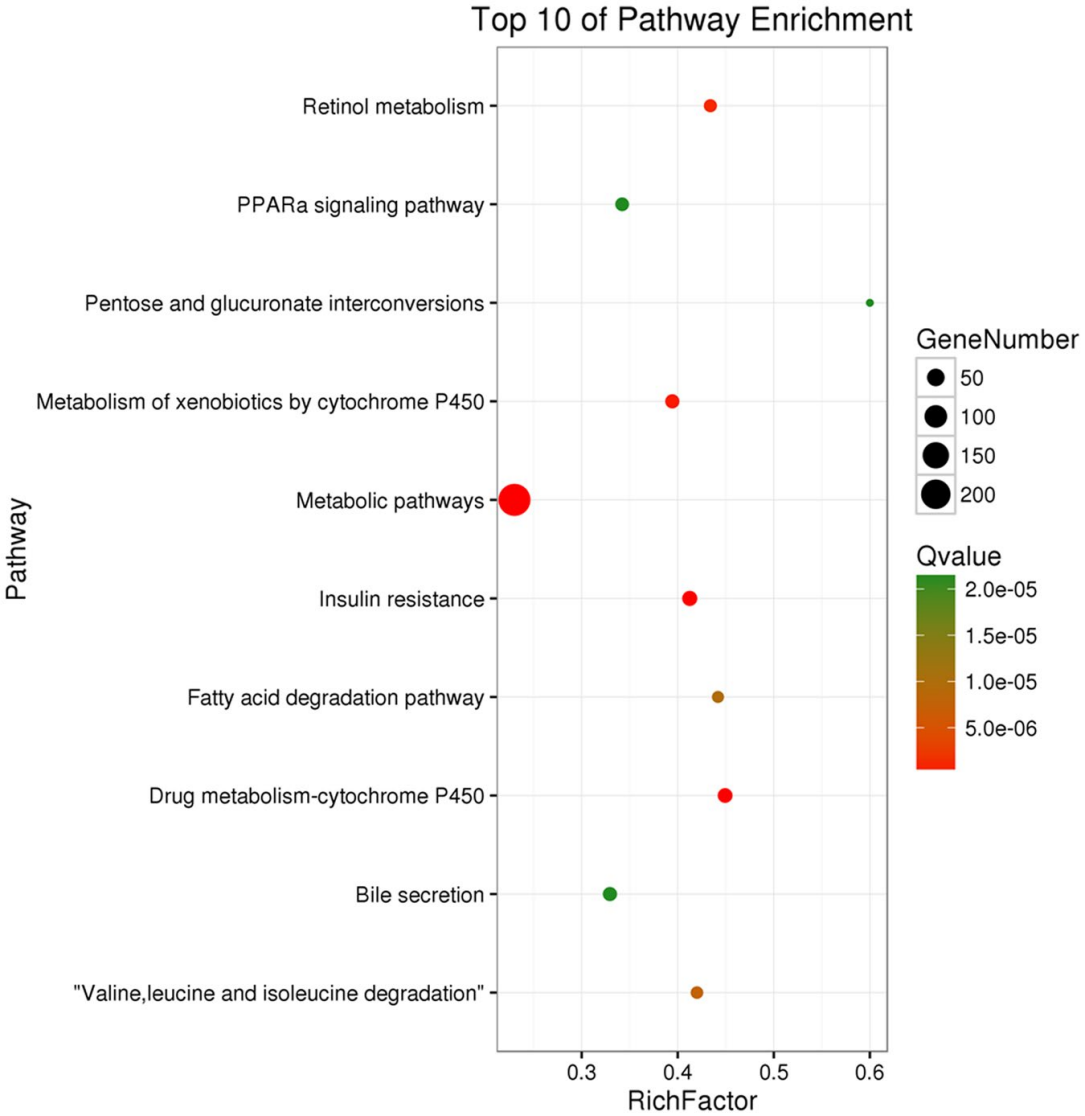
Scatter plot for KEGG enrichment results. The top 10 enrichment pathways are shown in the senior bubble chart. The Rich factor is the ratio of DEGs numbers annotated in this pathway term to all gene numbers annotated in this pathway term. A Q value is the corrected p value.

In order to explore the mechanism of fatty liver aggravated acute pancreatitis, we performed KEGG pathway analysis of the dysregulated genes between APFL and AP. The results indicated that fatty acid degradation pathway (ko00071) and PPARα signaling pathway (ko03320) may be involved in the pathogenesis of APFL. It was recognized that the disorder of lipid metabolism will aggravate the condition of the pancreatitis, so we choose these pathways to further analyze. The KEGG results of the top 10 pathways enrichment are shown in Fig. 5.

### Dysregulated genes participated in fatty acid degradation and PPARα signaling pathway

**Figure 6:**
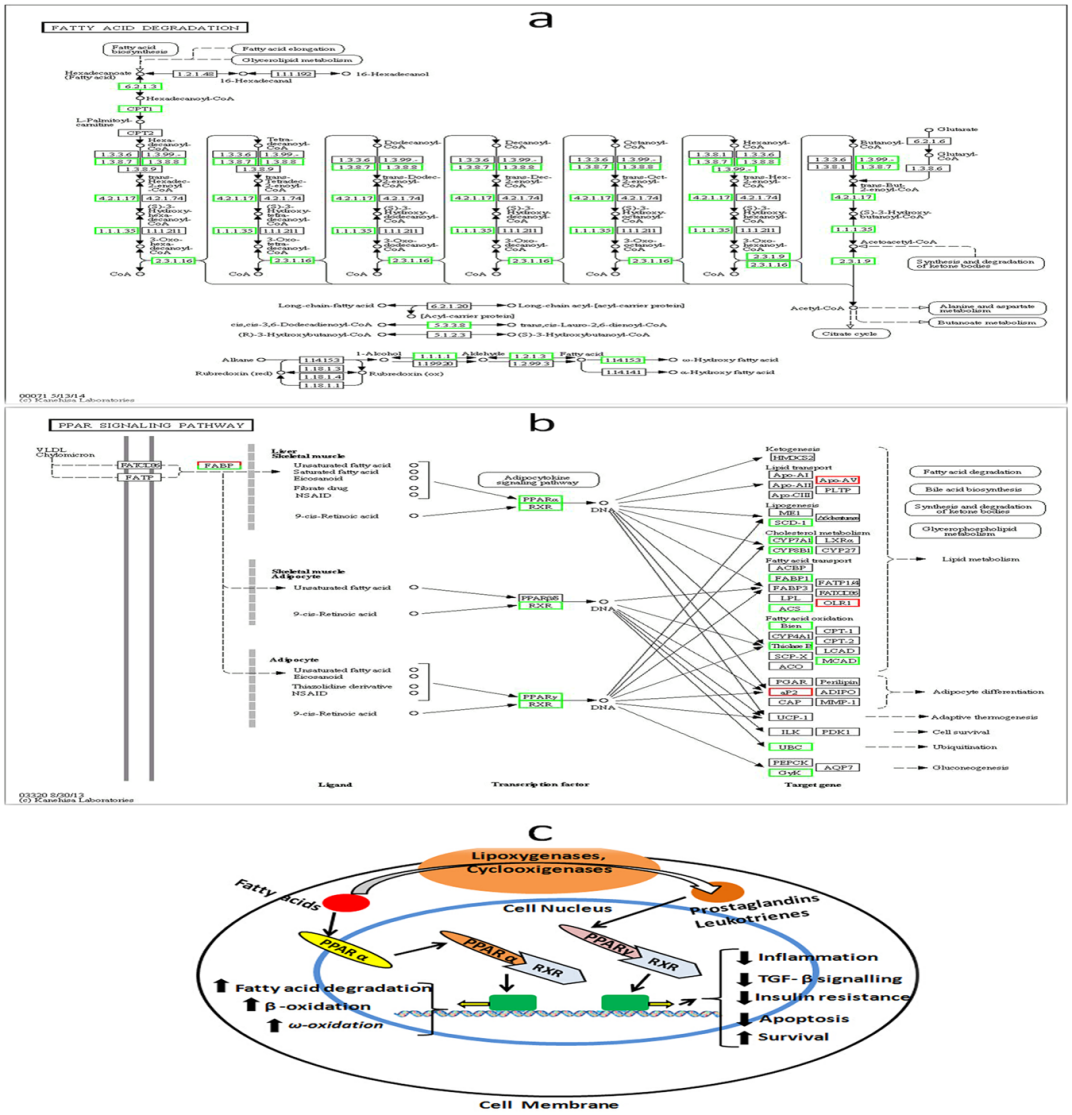
DEGs related to fatty acid degradation and PPARα signaling pathway between APFL and AP. KEGG pathway maps for (**a**) fatty acid degradation pathway (ko00071) and (**b**) PPARα signaling pathway (ko03320)^49^. Up-regulated genes are marked with red borders and down-regulated genes with green borders. Non-change genes are marked with black borders. Physiological function of the peroxisome proliferator activated receptors (PPARs) is shown in (**c**).

KEGG pathway analyses of the dysregulated genes between APFL and AP indicated that fatty acid degradation and PPARα signaling pathway may be involved in the pathological process of APFL. The detailed information about these pathways in KEGG database is shown in Fig. 6a,b. We found that most of the key genes involved in fatty acid degradation were significantly down-regulated, a reflection of lipid metabolic disorder. Meanwhile, some related genes in PPARα signaling pathway were also down-regulated, which would further aggravate lipid metabolism disorder. The physiological function of the PPARα signaling pathway in fatty acid degradation is illustrated in Fig. 6c.

### Gene expression cluster

**Figure 7:**
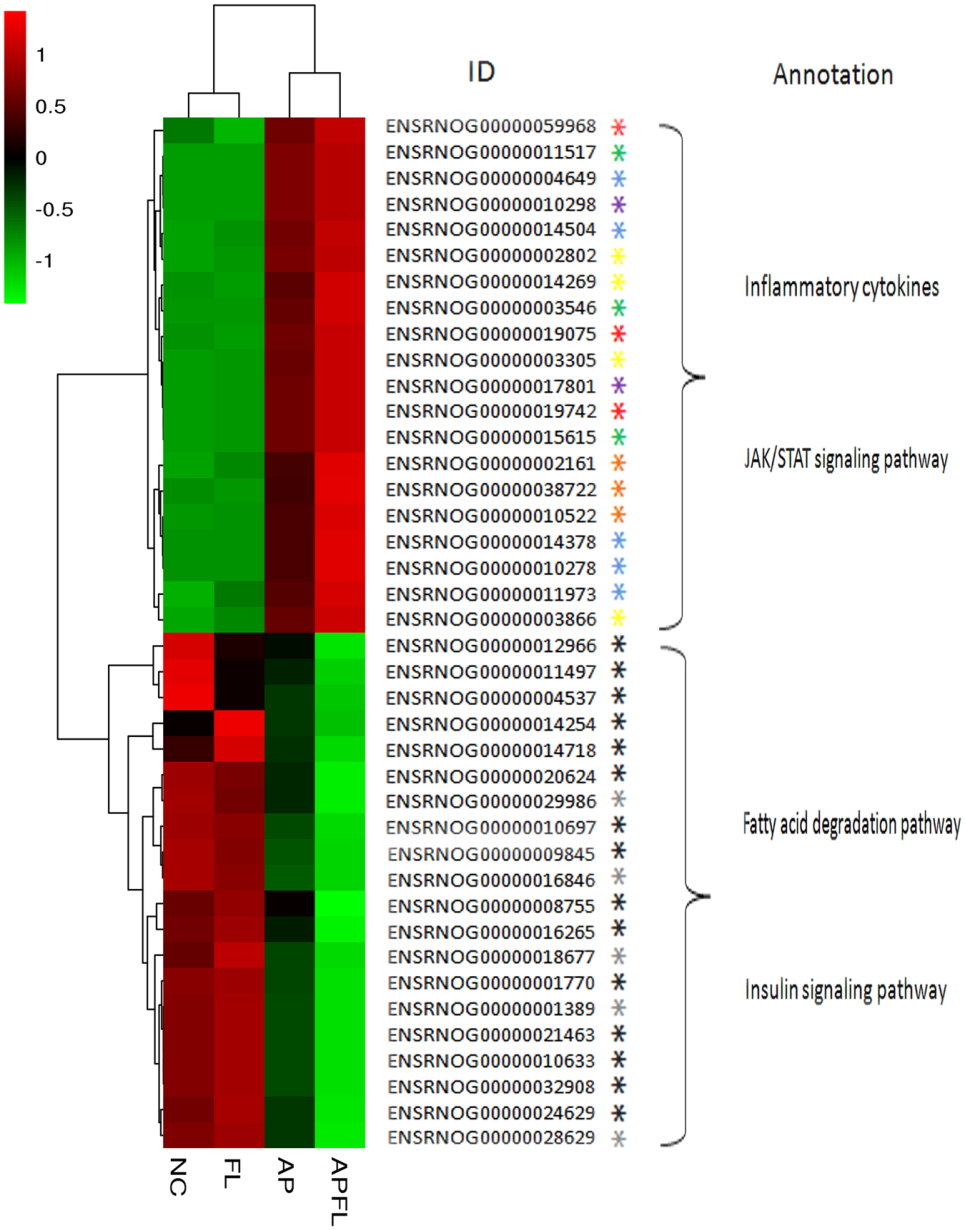
Cluster analysis of DEGs annotated in pathways associated with lipid metabolism and inflammation. The heatmap shows the expression levels of DEGs between APFL group and AP group. Transcript levels of genes encoding components involved in lipid metabolism were marked with an asterisk (*), insulin signaling pathway were marked with an asterisk (), JAK/STAT signaling pathway were marked with an asterisk (), endoplasmic reticulum stress were marked with an asterisk (), chemokine receptors were marked with an asterisk (), tumor necrosis factor receptor superfamily were marked with an asterisk (), interleukin were marked with an asterisk () and toll-like receptors were marked with an asterisk ().

A hierarchical cluster of DEGs is partially shown in Fig. 7. Compared with the AP group, a significant number of genes were up-regulated in APFL group encoding proteins linked to inflammatory processes, most prominently chemokines and chemokine receptors (e.g., CXCR2, CXCL1, CXCR4 and CCR1), and tumor necrosis factor receptor superfamily (e.g., TNFRSF21, TNFRSF12a and TNFRSF11a). This suggests that inflammatory reaction is more serious in the APFL group than in AP group. A large number of genes involved in lipid metabolism (e.g., ACADL, ALDH1B1, CPT1A, PPARα, ACADSB, ACSL5, ACSL3, HADH, ACADM, and ACSL1) were significantly down-regulated in APFL group compared with the AP group. This reflects that the fatty liver rats after induction of acute pancreatitis can appear more serious lipid metabolic disorder than non-fatty liver rats.

### Quantitative RT-PCR validation of the dysregulated genes that are involved in PPARα and fatty acid degradation pathway

**Figure 8:**
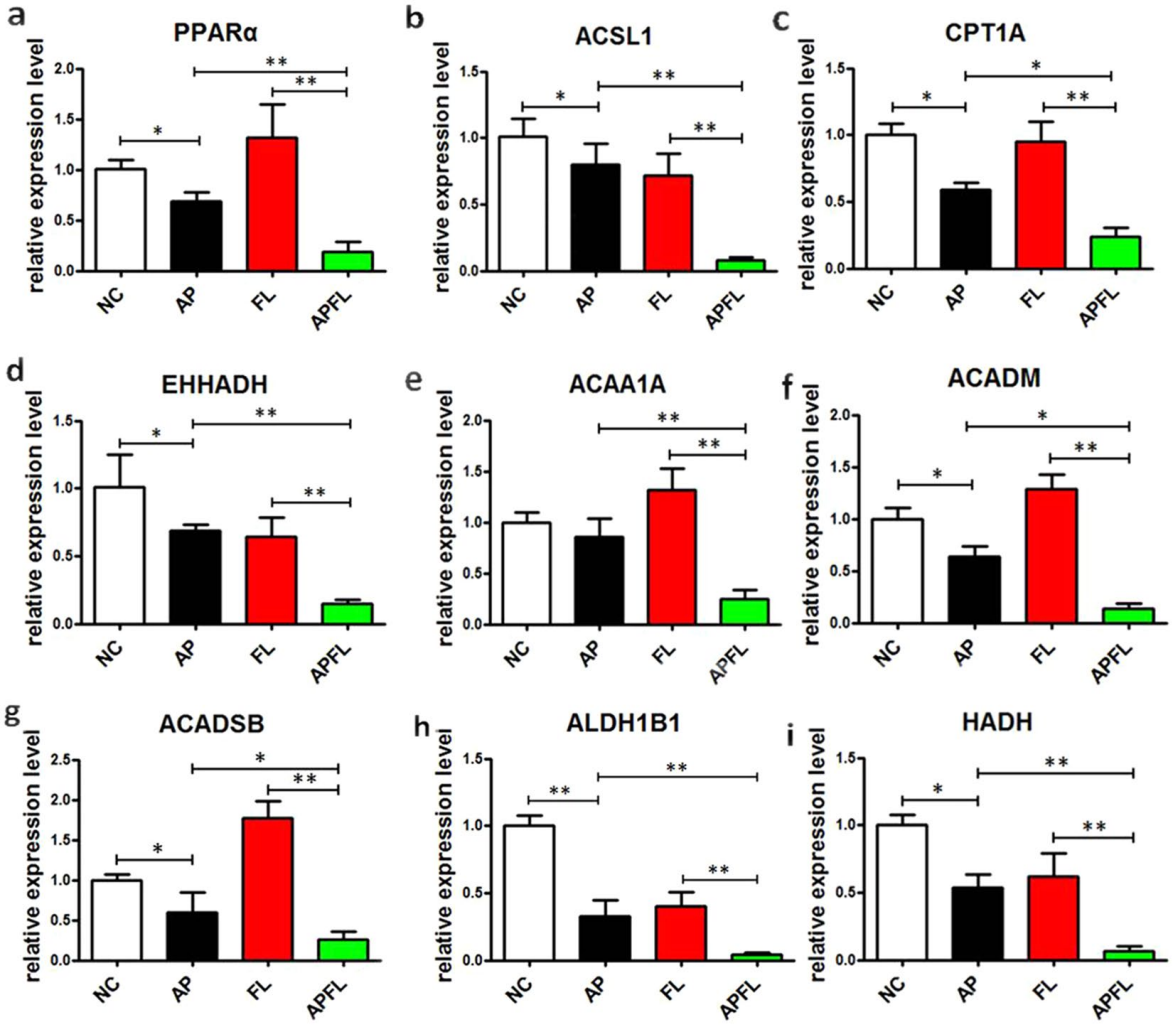
Quantitative RT-PCR validation of the selected dysregulated genes associated with fatty acid degradation. The expression levels of PPARα (**a**), ACSL1 (**b**), CPT1A (**c**), EHHADH (**d**), ACAA1A (**e**), ACADM (**f**), ACADSB (**g**), ALDH1B1 (**h**) and HADH (**i**) in NC, AP, FL and APFL were validated using qRT-PCR. The bar graph shows the expression of each gene in AP, FL, APFL relative to the average expression levels in NC. All error bars indicated S.D. *P < 0.05 vs NC, **P < 0.01 vs NC, ^#^ *P* < 0.05 vs FL.

The RNA-seq results of selected genes were validated by real-time RT-PCR. The dysregulated genes PPARα, ACSL1, CPT1A, EHHADH, ACAA1A, ACADM, ACADSB, ALDH1B1, HADH in mRNA expression profiling results were selected for qRT-PCR validation. The qRT-PCR results confirmed that the expression of these genes decreased after induction of pancreatitis, which was in agreement with the RNA-seq results. Compared with AP group, the expression levels of PPARα, ACSL1, CPT1A, EHHADH, ACAA1A, ACADM, ACADSB, ALDH1B1 and HADH in APFL group were significantly lower (Fig. 8).

### Coefficient analysis of fold change data between qRT-PCR and RNA-seq

**Figure 9:**
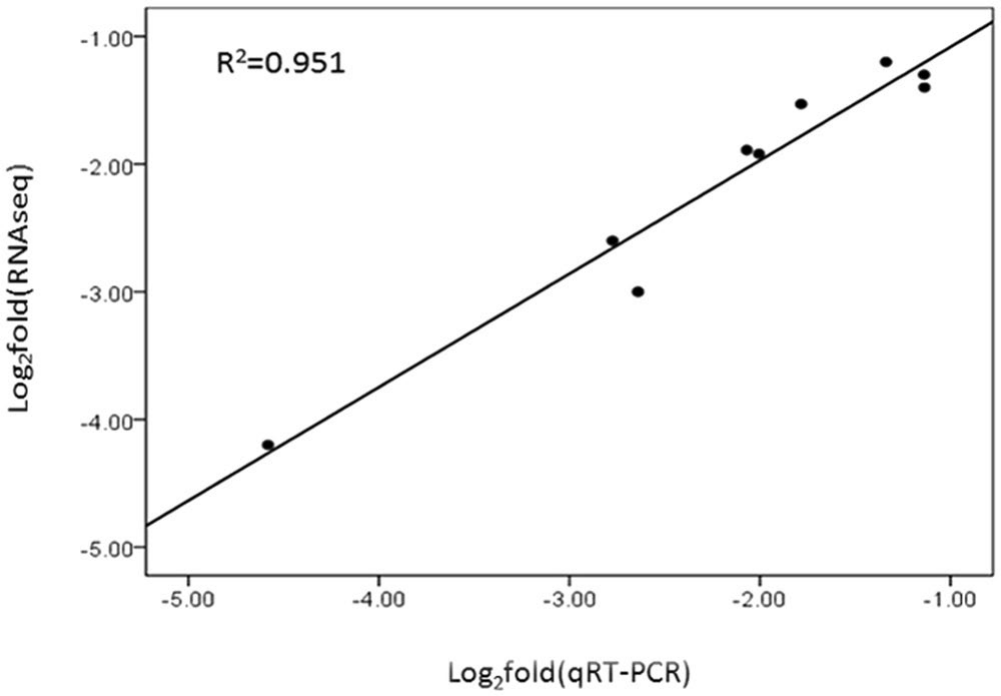
Linear regression analysis of fold change data between qRT-PCR and RNA-seq. Black dots represent log_2_ transformed fold change values of a single gene in APFL sample obtained from qRT-PCR (X-axis) and RNA-seq analysis (Y-axis). R: correlation coefficient.

Correlation analysis showed significantly positive correlation in fold change data between qRT-PCR and RNA-seq (a correlation coefficient R = 0.975), confirming our transcriptome analysis (Fig. 9).

## Discussion

Recent studies demonstrated that acute pancreatitis patients with fatty liver or obesity are at higher risk for developing severe acute pancreatitis (SAP) than non-fatty liver pancreatitis patients^9^. Moreover, the occurrence of systemic inflammatory response syndrome (SIRS)^14^, severe metabolic disorders (SMD) and acute respiratory distress syndrome (ARDS)^15^ is also significantly increased. However, if multiple lines of evidences proved that fatty liver disease is a negative prognostic factor for pancreatitis^16–19^, the precise mechanisms remain largely unknown. Therefore, it is of major importance to approach these mechanistic issues by comparing the pathophysiology of APFL and AP. It is why the present study compared the changes of gene expression between APFL and AP using RNA-seq method.

In this study, we found a large number of DEGs between APFL and AP groups. KEGG pathway analyses of these DEGs indicated that PPARα signalling pathway and fatty acid degradation pathway may be involved in the pathogenesis of APFL (Fig. 6). This provides clues that fatty liver may aggravate pancreatitis through the above pathways.

The Peroxisome Proliferator Activated Receptor alpha (PPARα) is a transcription factor belonging to the nuclear hormone receptors superfamily^20, 21^. Upon interaction with their ligands, such as unsaturated fatty acids (FA) and prostaglandins, PPARα translocates into the nucleus and dimerizes with the retinoid X receptor (RXR). Then, the complex triggers activation of target genes involved in fatty acid oxidation and other biological functions (Fig. 6c). PPARα plays an important role in metabolic regulation and affects the different links of lipid metabolism, including fatty acid uptake, fatty acid activation, intracellular fatty acid binding, mitochondrial and peroxisomal fatty acid oxidation^22^. In the present study, we found that the expression level of PPARα gene was significantly decreased in the APFL group compared with the AP group. It was interesting to note that the expression levels of many classical PPARα targets, including carnitine palmitoyl transferase 1a (CPT1A), enoyl-CoA, hydratase/3-hydroxyacyl CoA dehydrogenase (EHHADH), acyl-CoAsynthetase long-chain family member 1 (ACSL1) and acetyl-Coenzyme A acyltransferase 1A (ACAA1A), were also sharply decreased in the APFL group compared with the AP group (Fig. 8). Cpt1a is a protein that catalyzes the rate-limiting step of fatty acid β-oxidation^23^, and PPARα can stimulate acyl-CoA import into the mitochondria by increasing the expression of Cpt1a. Decreased expression of hepatic Cpt1a could reduce fatty acid catabolism but promote anabolic pathways thus resulting in lipid accumulation and hypertriglyceridemia^24^. Apart from CPT1A, EHHADH, an enzyme that is involved in peroxisomal oxidation of fatty acids^25^, was also significantly decreased in the APFL group compared with the AP group. EHHADH is a bifunctional enzyme, which carries both enoyl-CoA hydratase and 3-hydroxyacyl-CoA dehydrogenase activity^25^. Therefore, the decline in the expression of this enzyme will result in the disruption of peroxisomal β-oxidation of acyl-CoAs. Acyl-CoA synthetase activity is essential to convert fatty acids to their acyl-CoA derivatives. Some cytosolic acyl-CoA synthetases are under transcriptional control of PPARα, such as ACSL1 and ACSL5^26^. Furthermore, we found that the expression of ACSL1 was sharply decreased in the APFL group, which would further result in lipid metabolism disruption. Therefore, lipid metabolism pathway regulated by PPARα was inhibited in APFL rats.

In addition to some classic PPARα regulatory targets, the expression of other key enzymes in regulating fatty acid β-oxidation, such as acyl-Coenzyme A dehydrogenase (ACADM), short/branched chain acyl-CoA dehydrogenase (ACADSB) and hydroxyacyl-CoA dehydrogenase (HADH) have also been shown to be down-regulated in APFL group. Taken together, our results revealed that rats with fatty liver after induction of acute pancreatitis can appear more serious lipid metabolic disorder than non-fatty liver rats.

Over the past decade, several studies have confirmed that severe disturbance of lipid metabolism will aggravate acute pancreatitis processes^27, 28^. More specifically, excess free fatty acids cause oxidative stress, microcirculatory disturbance, free radical accumulation, and acinar necrosis in pancreatitis^29–32^. In addition, the interstitial release of triglyceride degradation products may exacerbate cellular disruption and increase inflammatory mediators, leading to systemic inflammatory response syndrome (SIRS) and organ failure. Nawaz *et al*.^33^ have found that elevated serum triglycerides (TG) are independently associated with persistent organ failure in acute pancreatitis patients. Zeng *et al*.^34^ proposed that disturbance of lipid metabolism might be a risk factor for respiratory failure. Wu *et al*.^35^ reported that lipid metabolism disorder is a risk factor for acute renal injury in acute pancreatitis patients. TG-mediated lipotoxicity promotes the development of mild pancreatitis to severe pancreatitis. Lipotoxicity therefore may be an attractive target to design novel interventions for severe acute pancreatitis^33^. In conclusion, the disturbance of lipid metabolism can indeed aggravate acute pancreatitis in many ways.

Our research has some guiding significance in clinical therapy. To our knowledge, this is the first study to further compare the gene expression profiles between APFL and AP by using RNA-seq method. We found that rats with fatty liver after induction of acute pancreatitis can appear more serious lipid metabolic disorder than non-fatty liver rats. Acute pancreatitis patients with severe degrees of hepatic steatosis may have a higher burden of lipid metabolic disorder, which would further aggravate the course of pancreatitis. So clinicians should be aware of this high-risk group and take effective measures to promptly correct lipid metabolism disorder in order to prevent the further development of the disease.

Previous studies have shown that inflammatory responses and pro-inflammatory cytokines are early up-regulated in acute pancreatitis and may exacerbate its severity. We also found that the gene expression of chemokines such as IL-1β, IL-6, IL1R1 and IL1R2 increased significantly in APFL group when compared with AP group (Fig. 7). These signaling molecules have been shown to play a pivotal role in the progression of experimental acute pancreatitis^1, 36, 37^. Moreover, the excessive activation of JAK/STAT signaling pathway and toll-like receptor signaling pathway was also found in APFL group as shown in heat map. JAK/STAT signaling pathway is involved in the regulation of many inflammatory responses^38^. Toll-like receptor signaling pathway belongs to innate immune responses, which plays an important role in pancreatitis^39, 40^. The over-activation of the above pathways in APFL group suggests that fatty liver may aggravate pancreatitis through JAK/STAT and Toll-like receptor signaling pathway.

**Figure 10:**
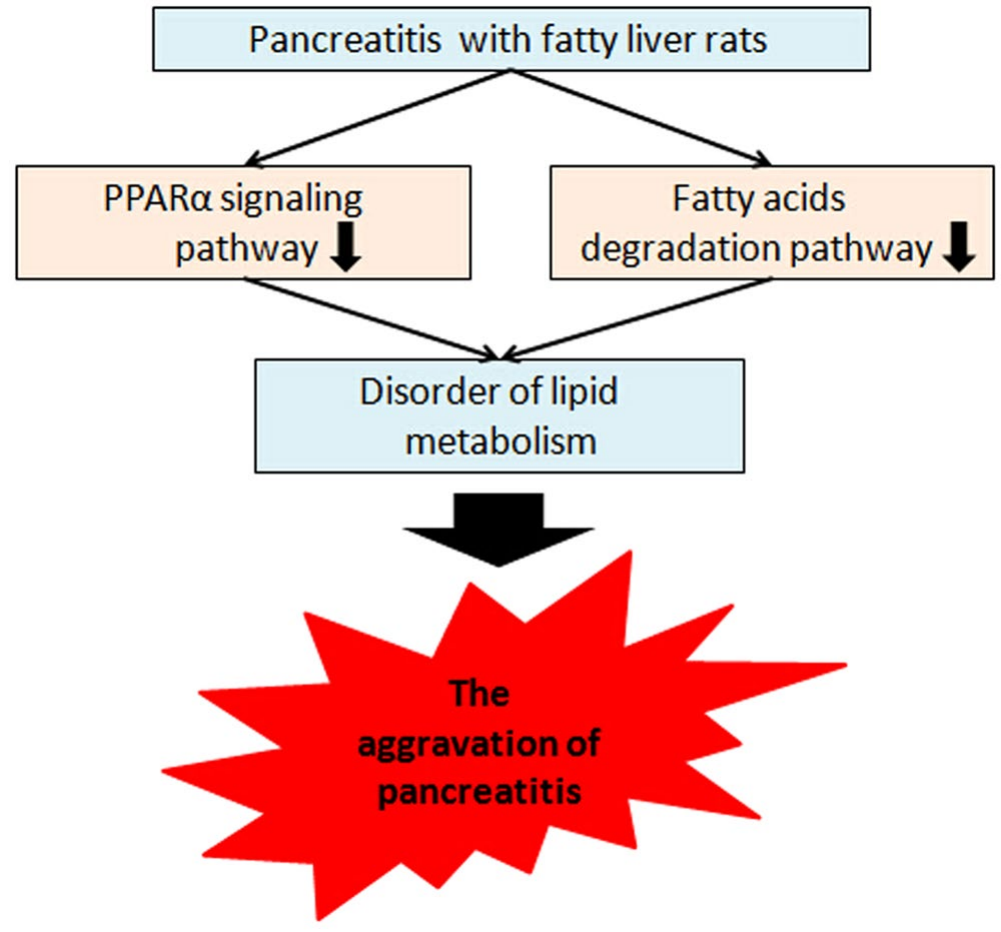
The schematic diagram of fatty liver aggravates pancreatitis. Fatty liver may aggravate pancreatitis by affecting lipid metabolism. The disorders of fatty acids degradation pathway and PPARα signaling pathway are involved in the course of APFL. Fatty liver may inhibit these two pathways to aggravate lipid metabolism disorder, which may further aggravate pancreatitis.

In conclusion, fatty liver can aggravate pancreatitis through a variety of mechanisms. In the present study, a significant number of differentially dysregulated genes were obtained by comparing the gene expression profiles of APFL and AP. Our study also provided the first evidence that the disorders of PPARα signaling pathway and fatty acids degradation pathway are involved in the course of APFL (Fig. 10), which sheds some new insight on our understanding of the pathophysiology of pancreatitis.

## Methods

### Experimental fatty liver model

The rat model of fatty liver was established by feeding a high fat diet. The high fat diet (HFD) group received the D12492 feed (Research Diets Inc.) and had 60% of their energy from fat, 20% from carbohydrates, and 20% from proteins. After two months on the HFD, the fatty liver rat model was established. The Normal diet group had 5% of their energy from fat, 76% from carbohydrates, and 19% from proteins. All rats were given free access to water and food.

### Experimental acute pancreatitis model

Ten-to-twelve week old Sprague-Dawley (SD) rats were obtained from the Animal Experimental Center of The Fourth Military Medical University (Xi’an, China). Acute pancreatitis (AP) was surgically induced as described previously^12^. Briefly, the rats were anesthetized via a peritoneal injection of 1% pentobarbital sodium (5 ml/kg). After exposure of the common bile duct and the pancreas, microaneurysm clips were placed on the bile duct. 5% sodium taurocholate (0.4 ml/kg, Sigma-Aldrich) was slowly infused into the common biliopancreatic duct. On completion of the infusion, the two microclips were removed. After ensuring that there was no bile leakage at the puncture level, the abdomen was closed in two layers. The entire procedure was performed using sterile techniques. All the procedures involving animals were reviewed and protocols were approved by Xijing Hospital Animal Care and Use Committee. All methods were performed in accordance with NIH guidelines.

### Histological assessment

The rats were sacrificed 8 h after the induction of AP. Liver and pancreas were collected for HE staining, which was performed as described previously^12^. Briefly, tissues were fixed in 4% neutral formalin for 24 h and embedded in paraffin to be cut into slices, which were stained by hematoxylin and eosin.

### Transcriptome analysis

The RNAseq technique was used in this study to analyze the gene expression profiling in acute experimental pancreatitis rats with and without fatty liver. The total RNA of liver samples was isolated using the Trizol Kit (Promega, USA). RNA quality was verified using Agilent 2100 Bio-analyzer (Agilent Technologies, Santa Clara, CA). The cDNA fragments were purified using a QIAquick PCR extraction kit following the manufacturer’s instructions. Then the cDNA fragments were enriched by PCR to construct the final cDNA library, which was sequenced on the Illumina sequencing platform (IlluminaHiSeq™ 2500).

### Transcript assembly and expression value estimation

All the clean reads were mapped to reference genome using TopHat^41^. Cufflinks package was used to estimate expression profile^42, 43^. Cufflinks were used to reconstruct transcript based on genome annotation, and then the transcripts were merged by cuffmerge. Finally, cuffquant and cuffnorm were used to estimate transcript expression.

### Differentially expressed genes (DEGs) and function enrichment analyses

DEGs were conducted using edger^44^. The false discovery rate (FDR) was used to determine the threshold of the p-value in multiple tests. A threshold of the FDR ≤ 0.05 was used to judge the significance of gene expression differences. In this study, we adopted WebGestalt (an online tool) to perform GO and KEGG analysis as described previously^45–49^. GO enrichment analysis of DEGs was calculated according to the following equation:

$${\rm{P}}=1-\sum _{i=0}^{m-1}\frac{(\begin{array}{c}M\\ i\end{array})(\begin{array}{c}N-M\\ n-i\end{array})}{(\begin{array}{c}N\\ n\end{array})}$$

N is the number of all genes with GO annotation; n is the number of DEGs in N; M is the number of all genes that are annotated to certain GO terms; m is the number of DEGs in M. The formula used in pathway analysis is the same as that used in GO analysis. N is the number of all genes with KEGG annotation, n is the number of DEGs in N, M is the number of all genes annotated to specific pathways, and m is the number of DEGs in M. The calculated p-value was adjusted using the Bonferroni correction, and the corrected p-value (Q-value) ≤ 0.05 was selected as the threshold. Fragments per kilobase of exon per million fragments mapped (FPKM) were calculated according to the following equation:

$${\rm{FPKM}}={10}^{9}({\rm{C}})/{\rm{NL}}$$

Given FPKM (X) is the expression of gene X, C is the number of reads uniquely aligned to gene X, L is the number of bases in gene X and N is the total number of reads uniquely aligned to all genes.

### Quantitative RT-PCR for mRNAs

Total RNA was extracted using RNA Extraction Kit (TaKaRa Biotechnology, Dalian, China) according to manufacturer’s instructions, and the concentration of the total RNA was quantified by measuring the absorbance at 260 nm. Five hundred ng RNA of each sample were subjected to cDNA synthesis using TaKaRa PrimeScript RT reagent kit (TaKaRa Biotechnology, Dalian, China). Quantitative real-time PCR was performed using SYBR Premix Ex Taq II (TaKaRa) and measured on a LightCycler 480 system (Roche, Basel, Switzerland). GAPDH was used as an internal control. The 2^−ΔΔCT^ method was used to calculate the relative expression levels of each gene. The sequences of the PCR primers are shown in Supplementary Table S1.

### Statistical analysbis

Heat map, senior bubble map, scatter plot map and venn map were performed using the OmicShare tools, a free online platform for data analysis (www.omicshare.com/tools). The Pearson correlation analysis was used to evaluate the fold change data between qRT-PCR and RNA-seq. Statistical analyses were performed using SPSS 17.0 software (IBM, Armonk, NY, USA). The Student t test was performed to examine the significance of differences between two groups. P-values less than 0.05 were considered statistically significant.

## Electronic supplementary material

Supplementary Information
