# Supplementary material for: RNA sequence analysis of rat acute experimental pancreatitis with and without fatty liver: a gene expression profiling comparative study
Source: Sci Rep. 2017 Apr 7;7:734. doi: 10.1038/s41598-017-00821-5 (PMC5429720; doi:10.1038/s41598-017-00821-5)
Supplement: Supplementary file 1 — Supplementary Information [file 41598_2017_821_MOESM1_ESM.pdf]

RNA sequence analysis of rat acute experimental pancreatitis with and without fatty liver: a gene expression profiling comparative study.

Qian Wang[1†], MD, Hongkai Yan[2†], MD, Gang Wang[1†], MD, Zhaoyan Qiu[3†], MD, Bin Bai[4], MD, Shiqi Wang[4], MD, Pengfei Yu[4], MD, Quanxin Feng[4], MD, Qingchuan Zhao[4*], MD, PHD, Xianli He[1*], MD, PHD, Chaoxu Liu[2,4*], MD.

[1]Department of General Surgery, Tangdu Hospital, Fourth Military Medical University, Xi'an 710038, China.

[2]Department of General Surgery, Huashan Hospital, Fudan University, Shanghai, 201907, China.

[3]Department of General Surgery, The General Hospital of the People's Liberation Army, Beijing, 100039, China.

[4]Department of Surgery, Xijing Hospital of Digestive Diseases, Fourth Military Medical University, Xian, 710032, China.

[†]These authors contributed equally to this study.

Running title: gene expression profiling of rat acute pancreatitis with fatty liver

[*]**Corresponding Author:**

Xianli He[1]

Qingchuan Zhao[2]

Chaoxu Liu[2,3]

[1]Department of General Surgery, Tangdu Hospital, Fourth Military Medical University, Xi'an 710038, China.

[2]Department of Surgery, Xijing Hospital of Digestive Diseases, Fourth Military Medical University, Xian, 710032, China.

[3]Department of General Surgery, Huashan Hospital North, Fudan University, No. 108 Luxiang Road, Shanghai, 201907, China

Tel: +86-21-66895079

Fax: +86-21-64085875

E-mail: [1]xianlihe@126.com, [2]zhaoqc@fmmu.edu.cn, [3]chaoxuliu@yahoo.com

Supplementary Figure S1

RNA sequencing quality assessment. Sequence reads quality assessment (a); Sequencing saturation analysis (b); Distribution of reads on reference genes of sample (c); Statistics of gene coverage (d); Correlations of expression values between replicates (e).

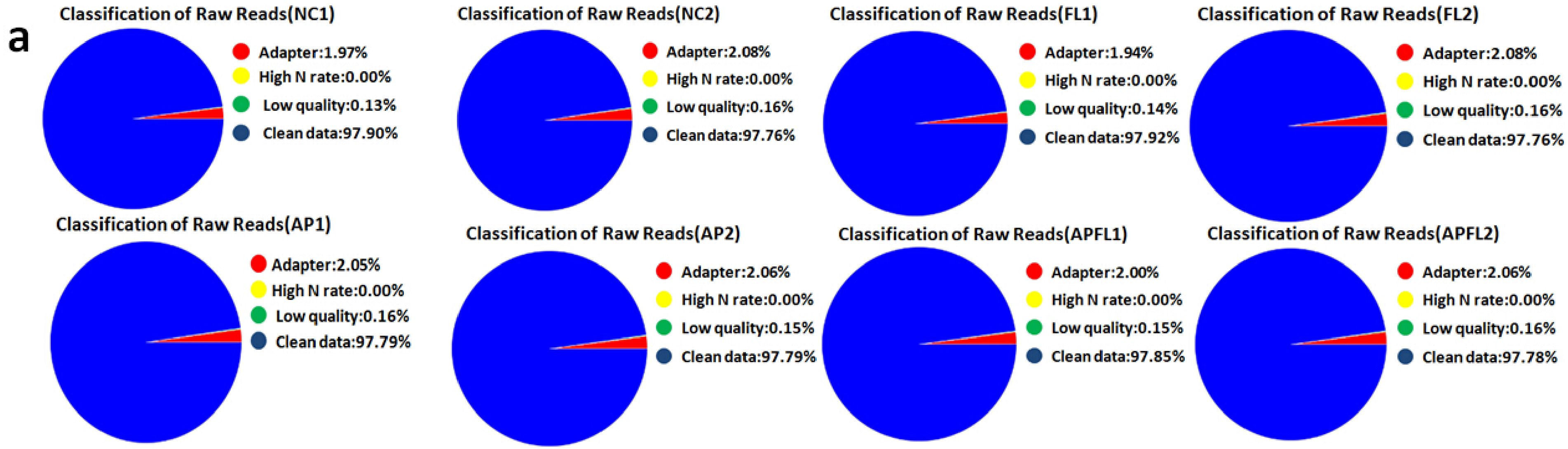

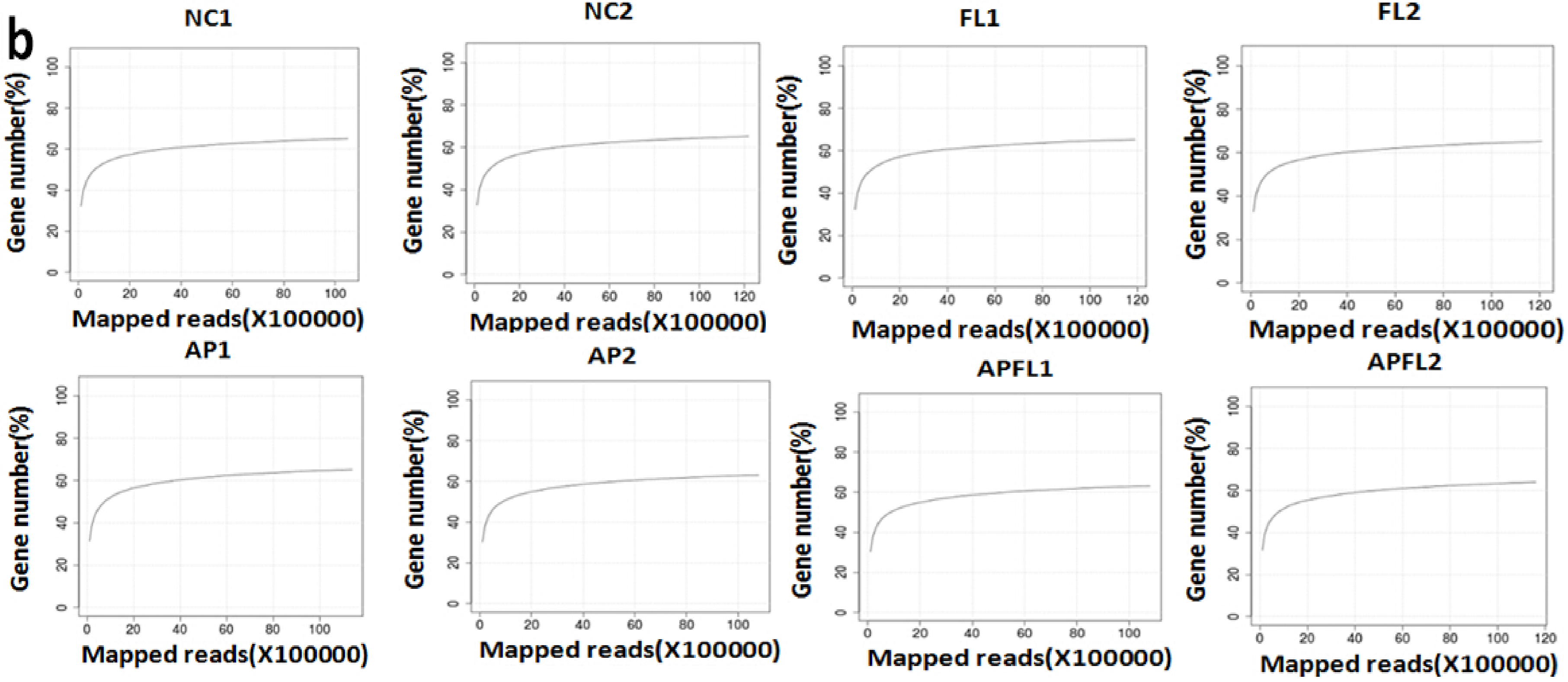
b
NC1
NC2
FL1
FL2
AP1
AP2
APFL1
APFL2
Gene number(%)
Mapped reads(X100000)

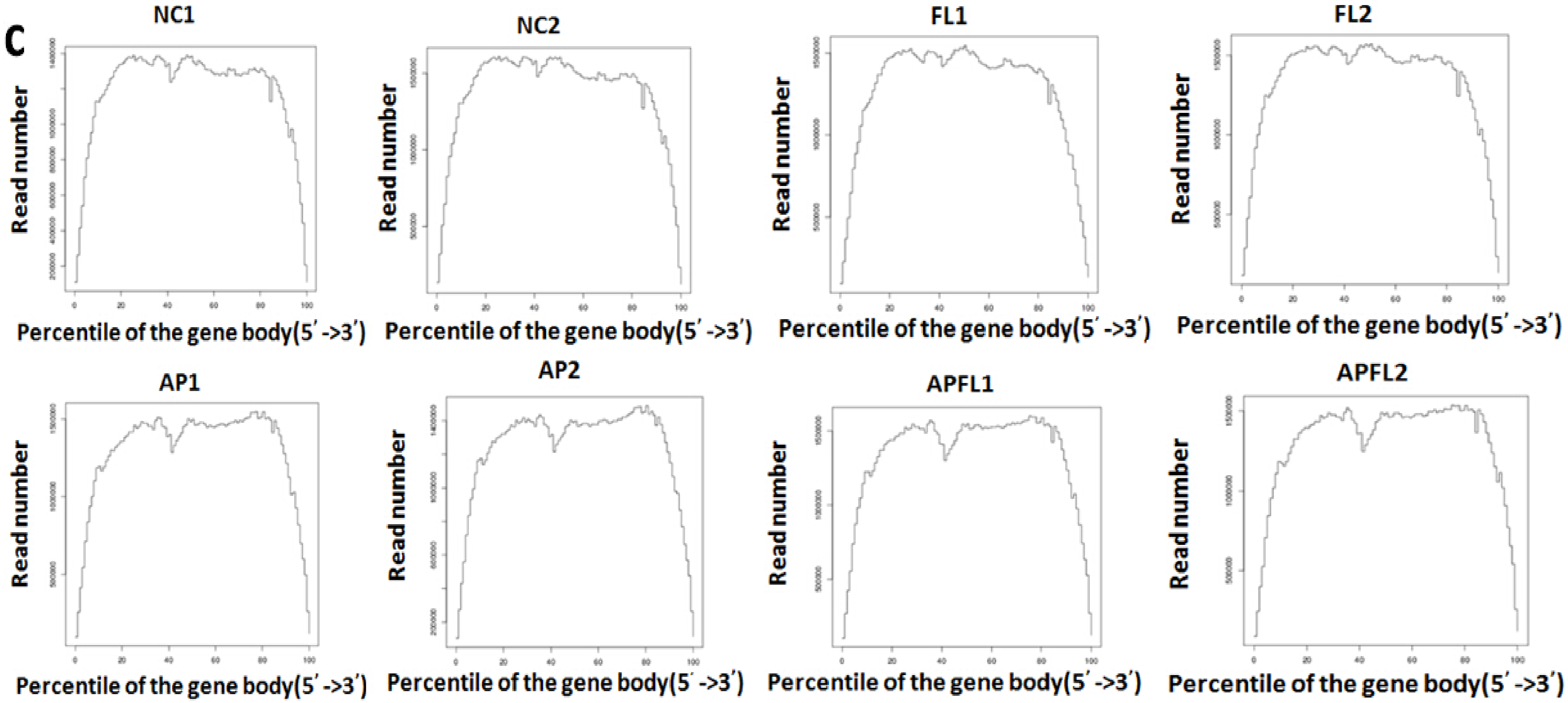
C
NC1
NC2
FL1
FL2
AP1
AP2
APFL1
APFL2
Read number
Percentile of the gene body(5′->3′)

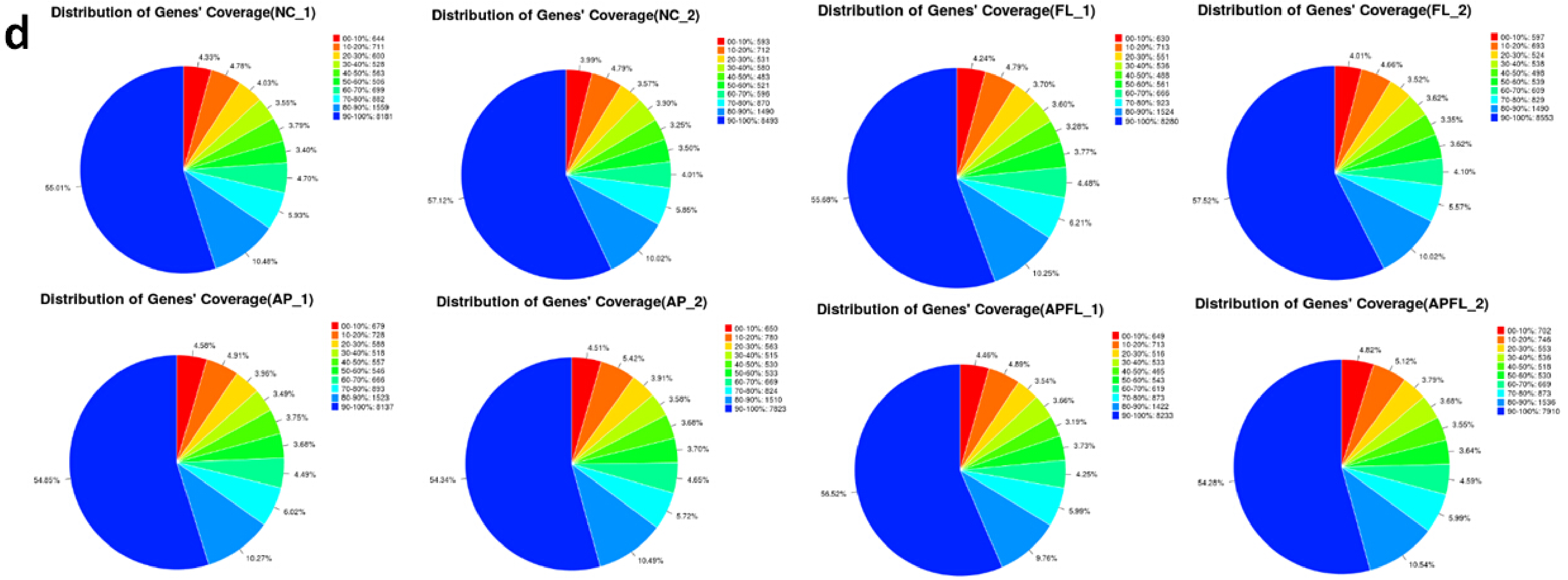
d
Distribution of Genes' Coverage(NC_1)
Distribution of Genes' Coverage(NC_2)
Distribution of Genes' Coverage(FL_1)
Distribution of Genes' Coverage(FL_2)
Distribution of Genes' Coverage(AP_1)
Distribution of Genes' Coverage(AP_2)
Distribution of Genes' Coverage(APFL_1)
Distribution of Genes' Coverage(APFL_2)

e

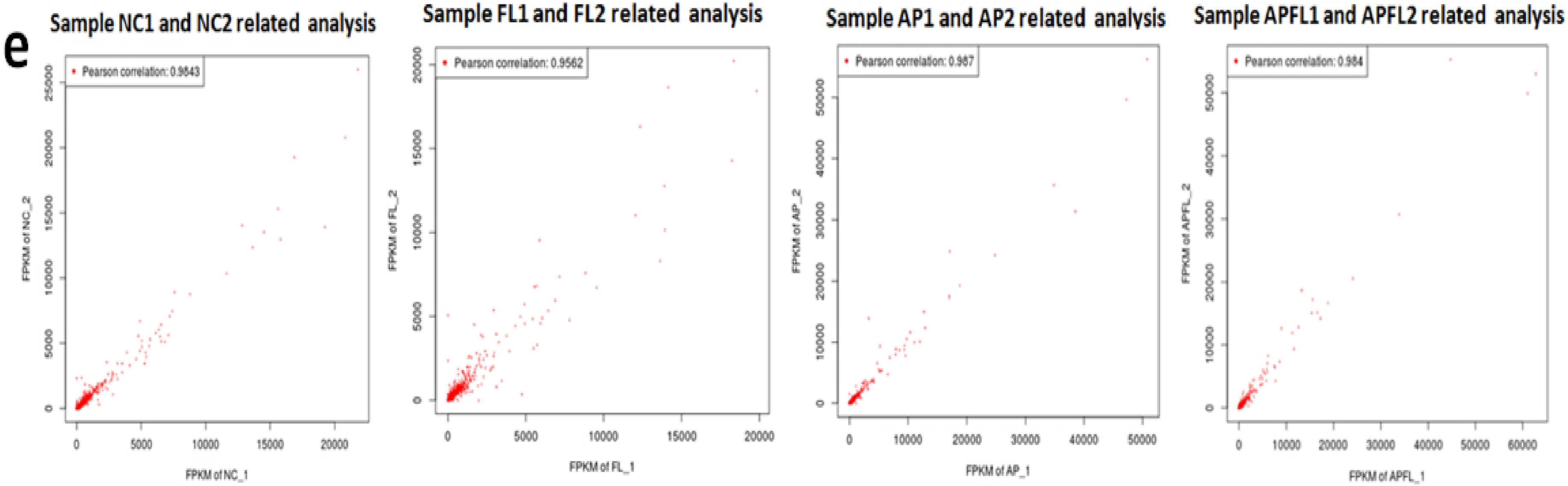
Sample NC1 and NC2 related analysis
Pearson correlation: 0.9843
FPKM of NC_1
FPKM of NC_2
Sample FL1 and FL2 related analysis
Pearson correlation: 0.9562
FPKM of FL_1
FPKM of FL_2
Sample AP1 and AP2 related analysis
Pearson correlation: 0.987
FPKM of AP_1
FPKM of AP_2
Sample APFL1 and APFL2 related analysis
Pearson correlation: 0.984
FPKM of APFL_1
FPKM of APFL_2

Supplementary Table S1

Supplementary Table S1 Primers for commonlydysregulated genes involved in PPAR and lipid metabolismfrom mRNA expression profiling results for quantitative RT-PCR validation.	
**Gene**	**Primer sequences(5'-3')**
PPARα	ForwardGGCAATGCACTGAACATCGAG
	ReverseGCCGAATAGTTCGCCGAAAG
ACSL1	ForwardTTCGCAGTGGCATCGTCAG
	Reverse TGTGATCATCAGCCGGACTTTC
CPT1A	Forward AGGTCGGAAGCCCATGTTGTA
	ReverseGCTGTCATGCGCTGGAAGTC
EHHADH	ForwardCTCGCCCAGAGCACATTGA
	ReverseACCCAACTGAGGCAGCATAGAAC
ACAA1A	ForwardGCATCTCACGGCAGAAGCAA
	Reverse TACGATCTCAGCACGGAAGCAG
ACADM	ForwardGCGTGACAGAACCCTCAGCA
	Reverse TCCACGATGAATCCGGTGAA
ACADSB	Forward AATGCCCTATGTTTCGCACCTC
	Reverse TGCTTCAACTTCAATGCCCATC
ALDH1B1	ForwardACATGGGTCAGTGCTGCTGTG
	ReverseACCTGAGGTCCCTGTTGAGTGTC
HADH	ForwardAGCACAGACCTGGTGGTGGA
	ReverseAGACGTGTTGCTGGCAAAGATG
